# Proteogenomics in cerebrospinal fluid and plasma reveals new biological fingerprint of cerebral small vessel disease

**DOI:** 10.1002/alz.086490

**Published:** 2025-01-09

**Authors:** Ilana Caro, Daniel Western, Yukinori Okada, Philip L. De Jager, Marie‐Gabrielle Duperron, Muralidharan Sargurupremraj, Sudha Seshadri, David Alexandre Tregouët, Carlos Cruchaga, Stéphanie Debette, neuroCHARGE

**Affiliations:** ^1^ Bordeaux Population Health Research Center, Inserm U1219, University of Bordeaux, Bordeaux, Bordeaux France; ^2^ NeuroGenomics & Informatics Center, Washington University School of Medicine, St. Louis, MO USA; ^3^ Department of Statistical Genetics, Osaka University Graduate School of Medicine, Suita Japan; ^4^ Center for Translational & Computational Neuroimmunology, Columbia University Irving Medical Center, New York, NY USA; ^5^ University of Bordeaux, Inserm, Bordeaux Population Health Research Center, UMR 1219, Bordeaux France; ^6^ UT Health San Antonio, Bordeaux, TX USA; ^7^ University of Texas Health Science Center at San Antonio, San Antonio, TX USA; ^8^ Glenn Biggs Institute for Alzheimer’s & Neurodegenerative Diseases, University of Texas Health Sciences Center at San Antonio, San Antonio, TX USA; ^9^ University of Bordeaux, Inserm, Bordeaux Population Health Research Center, Bordeaux France; ^10^ Washington University School of Medicine, Saint Louis, MO USA; ^11^ Bordeaux University Hospital, Department of Neurology, Institute of Neurodegenerative Diseases, Bordeaux France; ^12^ Bordeaux Population Health Research Center, Inserm U1219, University of Bordeaux, Bordeaux France

## Abstract

**Background:**

Cerebral small vessel disease (cSVD) is a leading cause of stroke and dementia. Its underlying mechanisms remain elusive and specific mechanism‐based drugs are lacking.

**Method:**

We integrated more than 2,800 CSF and 4,600 plasma pQTL, derived from the largest proteomic studies so far (SOMAscan 7k and 4k; in up to 35,559 individuals), and the two most prevalent MRI‐markers of cSVD (MRI‐cSVD, white matter hyperintensities and perivascular spaces burden; in up to 48,454 individuals) in a Mendelian Randomization (MR) framework to identify causal and druggable targets for cSVD. Identified association were followed‐up using a multipronged approach: across fluids, proteomics platforms (Olink 3072, N=8,590) and lifespan (N=1,748), using both MR and individual‐level data.

**Result:**

We found 51 proteins associated with MRI‐cSVD of which 46 in CSF and 9 in plasma. Among available significant CSF‐ and plasma‐proteins, 32% and 31% replicated in cross‐fluid and cross‐platform follow‐up, and 47% were associated with stroke and/or dementia at least at nominal significance. We found converging evidence that protein‐cSVD associations are enriched in extracellular matrix and immune response pathways. Immunity‐related proteins already showed association with MRI‐cSVD already in young adults in their twenties. Furthermore, we provide genetic support for drug repositioning opportunities for cSVD, including compounds crossing the blood brain barrier.

**Conclusion:**

Together, these findings provide a novel proteogenomic signature of cSVD and pave the way for novel therapeutic developments.

